# Ovarian Reserve Assessment for Infertility Investigation

**DOI:** 10.5402/2012/576385

**Published:** 2012-01-26

**Authors:** Bruno Ramalho de Carvalho, David Barreira Gomes Sobrinho, Andréa Duarte Damasceno Vieira, Manoela Porto Silva Resende, Antônio César Paes Barbosa, Adelino Amaral Silva, Hitomi Miura Nakagava

**Affiliations:** GENESIS-Centre for Assistance in Human Reproduction, SHLS 716, Bloco “L”, Salas “L” 328/331, Centro Clínico Sul, Ala Leste, 70.390 Brasília, DF, Brazil

## Abstract

The current trends to postpone motherhood and the increase in demand for assistance in reproductive medicine highlight the need for seeking guidelines for the establishment of individualized treatment protocols. Currently available ovarian reserve tests do not provide sufficient evidence to be solely considered ideal, but they may occupy important place in initial counseling, predicting unsatisfactory results that could be improved by individualized induction schemes and reducing excessive psychological and financial burdens, and adverse effects. In this paper, we revise the role of hormonal basal and dynamic tests, as well as ultrasonographic markers, as ovarian reserve markers, in order to provide embasement for propaedeutic strategies and their interpretation in order to have reproductive success.

## 1. Introduction

Considering modern trends of maternity postponement [1] and the increasing demand for assisted reproduction technologies (ART), the evaluation of functional ovarian reserve has arisen in an attempt to better advise interested couples, helping physicians in the inference of follicular response and success rates, and guiding the elaboration of individualized stimulation protocols, with a reduction of emotional and financial burdens of hard and stressful therapeutic processes. In this context, the identification of women with a lower reproductive potential is a great challenge for reproductive medicine specialists.

Sharara and Scott emphasized that an ideal ovarian reserve parameter should be easily measurable, minimally invasive, and inexpensive and should have good predictive values [2]. Serum and ultrasonographic markers have been tested to infer the gonadal reserve of infertile women, but none of them has been proven to confidentially reflect the complex follicular dynamics or to be strongly correlated with the size and/or quality of primordial follicles remaining in the gonads after each wave of follicular growth. In other words, those tests do not ideally reflect the pool of unrecruited follicles, which may be responsible for the continuity of ovulatory cycles and, therefore, for the long-term reproductive potential [3–7].

In this paper, we present a review of the literature, providing a rationale on the applicability of age and menstrual pattern as clinical markers, and the most commonly used tests for the evaluation of ovarian reserve in infertile women. Tests were divided into static (endocrine and ultrasonographic tests performed in the early follicular phase) and dynamic (endocrine tests assessing ovarian response to exogenous gonadotropic stimulus).

## 2. Clinical Markers

### 2.1. Age

Age is considered to be the single most important factor in determining quality and quantity of ovarian reserve. It is well known that both the quantity and quality of ovarian follicles significantly decrease as a woman advances in age and that many women who postpone maternity may be infertile at the time they are willing to become pregnant. Fecundability significantly declines since the early 30s [8], and the prevalence of infertility increases significantly after the age of 35 years; about 99% of patients are expected to be infertile with 45 years of age [9].

Fertility decline can be attributed to numerous events associated with advancing age, including changes in oocyte quality, frequency and efficiency of ovulation, sexual function, uterine diseases, and the risk of pregnancy complications, such as gestational diabetes and hypertensive disease. Also, genetic factors, smoking, infections, and adnexal surgeries shall be determinants of diminished ovarian reserve in older women [10].

In historical cohorts, the rates of infertility among women who were married at the age groups of 20–24, 25–29, 30–34, 35–39, and 40–44 years were 6%, 9%, 15%, 30%, and 64%, respectively [9]. Likewise, the probabilities of clinical pregnancy after intercourse on fertile days in women age groups of 19–26, 27–34, and 35–39 years were approximately 50%, 40%, and 30%, respectively, with partners in the same age groups [11].

Women undergoing artificial insemination with donor sperm because of male factor for infertility (azoospermia) presented with lower rates of conception or needed more cycles to achieve it after 35 years of age [12]. Similar results were obtained in *in vitro *fertilization (IVF) cycles using nondonor fresh eggs; birth rates for age groups of <35, 35–37, 38–40, 41-42, and >42 years were 36%, 28%, 18%, 10%, and 4% per cycle, respectively [13]. Data from *Red Latinoamericana de Reproducción Asistida* demonstrated a significant reduction of pregnancy rates per IVF cycle with follicular aspiration: 38% in women 30–34 years, 31% in women 35–39 years, and 16% in older patients [14]. Furthermore, studies have shown that women of advanced maternal age unable to achieve pregnancy through IVF are able to conceive using donor oocytes from younger women [15].

We have recently reinforced previous studies conclusions that ovarian response to exogenous stimulus for ART is significantly associated with age and, also, its capacity to predict the occurrence of poor response and pregnancy in intracytoplasmic sperm injection (ICSI) cycles. In our study, the number of preovulatory dominant follicles and the total number of oocytes and mature oocytes yielded were significantly higher between women with ≤35 years of age [16].

### 2.2. Menstrual Pattern

Menstrual cycle length (MCL) is supposed to be primarily determined by the rate and quality of follicular growth, and, thus, the duration of the follicular phase; in general, menstrual cycles range from 23 to 32 days [17] and exhibit a mean interval of 28 days. This pattern is commonly expected to be fairly persistent until the late 40s, but a gradual shortening in cycle length may initiate in the late 30s, in parallel with higher serum levels of follicular stimulating hormone (FSH) and lower serum levels of inhibins [18].

The age-dependent shortening of MCL is suspected to be related with shortened follicular phases; there is evidence that the mechanism behind it should, in part, involve diminished production of inhibin-B by a small number of antral follicles and a consequent precocious increase of FSH secretion [19, 20]. Such a hypothesis gains force from the demonstrated correlation between MCL and the antral follicle count (AFC) during ultrasonographic evaluation [21].

According to the study of Brodin et al., conception rate was significantly reduced among women with MCL shorter than 30 days and the adjustment for other potential predictors of fertility, like age, had not appreciably changed such an effect. Also, MCL had significant association with ovarian response to gonadotropin stimulus and embryo quality in IVF/ICSI cycles, and, even if the interference of age is excluded, pregnancy rates are almost twice as high among women with cycles >34 days when compared with those with cycles <26 days [21].

## 3. Endocrine Static Markers

### 3.1. Follicle-Stimulating Hormone

Early follicular phase (basal) FSH is the most studied and used endocrine test in determining ovarian reserve [6, 22]. Historically, combination of basal FSH and age was found to be better than age alone in predicting IVF outcome. Many IVF centers, then, continue to rely on basal FSH serum measurements, notwithstanding limitations like great variability in serum FSH levels within and between menstrual cycles, interference of external factors such as smoking [23], and disparities between assays [24].

van der Steeg et al. studied predictive value of basal FSH for spontaneous pregnancy occurrence in ovulatory subfertile women younger than 40 years and observed reduced chances when the levels exceeded 8 IU/L, whereas no association could be determined for lower levels [25]. In a recent prospective study, Haadsma et al. pointed basal FSH and AFC as significant predictors of spontaneous pregnancy [26].

The accuracy of FSH in predicting poor response to ART stimulation has been considered to depend on the identification of very high serum thresholds [6]. Including 212 patients submitted to IVF cycles, Ashrafi et al. observed that women with FSH levels ≥15 IU/mL had fewer aspirated oocytes and a larger number of cancelled cycles than women with lower levels, with no significant difference in gonadotropin doses administered [27]. Creus et al. also demonstrated mean FSH serum concentrations to be significantly higher among patients with cancelled ART cycles, which were independent from other variables, such as age and inhibin-B [28]. In the study of Al-Azemi et al., FSH was a good predictor of poor oocyte yield and only AMH presented a better performance for that purpose [24].

Regarding pregnancy occurrence, Klinkert et al. suggested that it was less frequent among women with FSH levels ≥15 IU/L when compared to those with lower levels, but with no statistical significance [29]. Also, it was recently demonstrated that pregnancy rates in women aged <35 years with elevated basal FSH were higher than those of older women with normal levels of the hormone [30], reinforcing age as a main ovarian reserve marker. In a previous study, van Montfrans et al. had already suggested that determination of basal FSH should not be decisive to the initial management of infertile women with clinically normal cycles, once pregnancy occurred in about half the women with elevated serum levels [31].

FSH continues to be an interesting test on ovarian reserve investigation, since it is an easily accessible and low-cost marker, and that could be useful in pretreatment evaluation of specific groups of infertile women, such as those carrying anovulatory cycles [31], endometriosis [32] or in patients over 35 years of age [33]. As it was recommended by Luna et al. [30], we believe that more careful counseling shall be provided for patients aged ≥35 years, who are more susceptible to lower ovarian response to stimulation and higher rates of ART cancellation. That said, high FSH levels should not be used to exclude women from proceeding with ART once adequately counseled.

### 3.2. Inhibin-B

Inhibins are glycoprotein hormones of the superfamily of transforming growth factors *β* (TGF-*β*) secreted by granulosa and theca cells [34]. Inhibin-B inhibits pituitary FSH secretion [35] and paracrine action on developing follicles, stimulated by the association of FSH itself with insulin-like growth factor I [36, 37]. Investigation on infertile patients 24–40 years of age demonstrated significant negative correlation between basal inhibin-B and FSH, and significant positive correlation between basal inhibin-B and AFC [38].

In accordance with that, Seifer et al. demonstrated that women with declining ovarian responsiveness and poor outcomes in ART presented with diminished basal serum concentrations of inhibin-B, in spite of nonelevated day 3 FSH levels. Furthermore, greater estrogen responses and higher amounts of oocytes were obtained after stimulation of women with serum inhibin-B levels ≥45 pg/mL, whereas cancellations were three times more frequent among those with lower levels [39]. Significant correlations were either found between the number of oocytes retrieved and serum inhibin-B concentration, and all patients with serum inhibin-B levels >100 pg/mL had >6 oocytes yielded [40]. In the study of Peñarrubia et al., day 5 inhibin-B was associated with live-birth rates after IVF with 68.71% sensitivity and 88.51% specificity, which was statistically significant and stronger than any other variable investigated [41].

Other studies, however, did not recommend the use of inhibin-B alone as a reliable predictor of ovarian reserve [42, 43]. McIlveen et al. observed significant intercycle variability in inhibin-B concentrations [44]. The systematic review of Broekmans et al. [6] was emphatic in counseling clinicians to be aware of the high rate of false positives in routine determination of basal inhibin-B and cautiously analyze exclusion of patients from IVF programs with base on its levels. Those authors stated that even using very low inhibin-B levels, accuracy of the test in predicting poor response was only modest when compared with the other available markers, even though it can be used as a tool for counseling infertile couples.

### 3.3. Estradiol

Basal estradiol (E2) levels may provide additional useful information for the evaluation of ovarian reserve. Early elevations in serum E2 are understood as a consequence of the advanced follicular development and early selection of a dominant follicle observed in older cycling women that are driven by rising FSH levels [18]. Higher rates of cancelled ART cycles have been demonstrated with E2 levels <20 pg/mL or ≥80 pg/mL [45]. Measurement of both FSH and E2 on cycle day 3 may, thus, help to diminish the incidence of false-negative tests based on measurement of FSH alone; when both markers are precociously elevated, poor ovarian response is likely to occur [18].

However, other studies favorable to the use of E2 as an ovarian reserve marker have been incapable to correlate it to follicular development or demonstrate its ability to predict the occurrence of pregnancy [45–48]. Fiçicioglu et al. have also demonstrated similar basal E2 levels in serum of good and poor responders to gonadotropic stimulus in ART cycles [49].

E2 may be used to guide the clinician as to whether the stimulation with gonadotropins can be started, but it does not have value as IVF prognostic tool. Considering the low predictive accuracy and the lack of high sensitivity and specificity cutoff levels [6], its role as a determinant for couples inclusion in ART programs should, therefore, be evaded.

### 3.4. Anti-Müllerian Hormone

Anti-Müllerian hormone (AMH), also referred to as Müllerian-inhibiting substance, is a glycoprotein hormone of the TGF-*β* superfamily expressed by granulosa cells as soon as the primordial follicles are recruited [50]; its expression is maintained until the follicles reach about 6 mm in diameter, when preantral/initial antral follicles are selected for dominance [51] and follicular growth follows controlled by FSH action [52].

The biological activity of AMH in women is not completely understood, but data along the last years suggest that it may act as a modulator of follicle recruitment and a regulator of ovarian steroidogenesis [50, 53]. AMH is known to have an inhibitory effect on the pool of primordial follicles, acting on pregranulosa cells in order to limit the number of recruitable follicular units and, later, as a decisive factor in permitting the FSH-dependent growth of ovarian follicles [52–56].

From the foregoing provisions, AMH determination has been proposed in clinical practice for the prediction of ovarian reserve. AMH is considered to be a marker that can estimate the quantity and activity of retrievable follicles in early stages of maturation, thus being more reliable for the prediction of ovarian response and reproductive potential [43, 50, 52, 57–60]. Other studies also supported this affirmative by demonstration of a decreasing rate in circulating AMH levels with age [61–63].

Compared to FSH, inhibin-B, and E2, AMH has the advantage of reduced variability of its serum concentrations along the menstrual cycle [5, 64, 65] and consequent malleability regarding the time of its determination. Mean values of 1.4 ± 1.1 ng/mL, 1.43 ± 1.08 ng/mL, and 1.35 ± 1.02 ng/mL were measured in follicular, periovulatory, and midluteal phases, respectively [64]. AMH stable serum levels were also demonstrated by other studies [52–54]; Tsepelidis et al. obtained a mean of 2.4 ± 1.1 ng/mL along the menstrual cycle in normoovulatory patients [66]. The reproducibility of AMH between cycles was also demonstrated; lower variations in serum levels between consecutive cycles were found for AMH when compared with those detected for FSH, inhibin-B, and E2, or the antral follicle count (AFC), among infertile women aged 20 to 40 years old [5].

In ART, van Rooij et al. detected reduced preinduction AMH levels and AFC in poor responders (women with canceled cycles or from whom fewer than four oocytes were yielded) compared to women with a good response to the exogenous stimulus. There was also a strong correlation between the two markers, who were found to be important predictors of the response [57]. Recent data were obtained in order to evaluate the efficacy of AMH levels and AFC to predict IVF outcomes and demonstrated that AMH is at least as efficient as AFC to predict high ovarian response, with regard to its ability to identify poor responders [67–69].

In a prospective evaluation of previous poor responders (fewer than 4 follicles with a diameter >15 mm) older than 38 years with a basal FSH >10 UI/L, AMH was considered to be the best single marker of the ovarian response to the exogenous stimulus, once patients with incomplete ART cycles presented with significantly reduced serum levels [70]. In the study by Fiçicioglu et al., serum AMH levels were significantly higher in women with adequate ovarian response to exogenous stimulus (0.67 ± 0.41 pg/mL) compared with poor responders (0.15 ± 0.11 pg/mL) [49], supporting the results of previous studies [57, 64]. In the prediction of low number of oocytes yielded, basal AMH presented the largest area under the curve (AUC = 0.92) among all variables tested, followed by AFC (AUC = 0.78) and age (AUC = 0.63) [49].

Fadini et al. confirmed AMH serum concentration as a highly effective predictor of the number of retrieved oocytes and, then, instrumental for the recognition of women with reduced ovarian function and those suitable for an oocyte *in vitro* maturation (IVM) treatment. Actually, AMH allowed authors to identify cycles able to produce satisfactory amounts of oocytes and, then, reduce the efficiency gap between IVM and conventional IVF [71].

The lack of a consensual cutoff combining satisfactory sensitivity and specificity for routine use continues to represent a considerable drawback. Muttukrishna et al. demonstrated 87% sensitivity and 64% specificity of AMH in the prediction of response and cancellation in ART, for a cutoff of 0.2 ng/mL [60]. La Marca et al. ratified the use of AMH for the prediction of a poor response by demonstrating 80% sensitivity and 93% specificity in AR cycles when a threshold of 0.75 ng/mL was considered [72]. An interesting study reported AMH value of 1.26 ng/mL by which 88% of poor responders should be identified (patients with less than 5 oocytes retrieved) in standard IVF cycles [73], and a similar cutoff (1.28 ng/mL) was determined as a guidance to decide the appropriateness of oocyte IVM as a treatment (at least 5 oocytes retrieved) [71].

Irez et al. have recently demonstrated the significant association between basal AMH serum concentration with the total number of oocytes and AFC, inhibin-B and FSH levels, oocyte quality and embryo development in intracytoplasmic sperm injection (ICSI) cycles. Also, the authors observed that early embryo cleavage rate was significantly higher among patients with AMH serum levels between 1.40 ng/mL and 4.83 ng/mL [74], supporting the hypothetical association of AMH with implantation rates [75–77]. Likewise, Arabzadeh et al. have recently demonstrated positive correlation between serum basal AMH levels and the number of oocytes retrieved and the percentage of mature oocytes, but significance was also not reached in correlation between the marker and implantation rate [78].

Attempts have also been made to correlate serum AMH levels with the occurrence of pregnancy following ART. Values higher than 2.7 ng/mL were associated with higher rates of implantation and pregnancy (although not significant), and the levels measured on the day of human chorionic gonadotropin (hCG) administration were superior (AUC = 0.647) to those of basal FSH regarding the prediction of embryo quality [22]. Recent studies carried out to determine the value of AMH as a marker of the response to the stimulus were unable to establish its value as a predictor of pregnancy [48, 73].

Finally, a recent systematic review pointed AMH as a useful test in the prediction of poor response and cycle cancellation and also of ovarian hyperstimulation in ART. The authors found it to be better than patient's age in predicting ovarian response to exogenous stimulus, with similar performance only for AFC [79].

Based on the favorable results reported and on the proven significant correlation with important variables such as AFC and the quantity of matured or aspirated oocytes, we believe in a promising future for AMH as an important clinical marker in the assessment of female infertility. At this moment, poor response can be associated with AMH serum levels <1 ng/mL, normal response with levels from 1 to 4 ng/mL, and high response with levels >4 ng/mL [60, 69, 72, 79, 80].

## 4. Endocrine Dynamic Tests

### 4.1. Clomiphene Citrate Challenge Test

Clomiphene citrate challenge test (CCCT) was first described by Navot et al. In the original study, 100 mg clomiphene citrate was administered to women aged ≥35 years from days 5–9 of the menstrual cycle. FSH, LH, and E2 levels were primarily determined on cycle days 2-3, and response was determined on cycle days 9–11. Diminished ovarian reserve was determined by day 3 FSH >14.9 mIU/mL or day 10 FSH > day 3 FSH [81].

CCCT was found to predict the occurrence of less than 6 aspirated oocytes in IVF cycles, with an expressive area under the ROC curve (AUC = 0.88) among women aged 18 to 39 years [82]. Years before, Corson et al. had associated prognostic CCCT value in determining response of women >35 years of age or those with previous poor response, although weak correlations with other tests limited conclusions [42]. In contrast, previous data had shown a higher sensitivity of basal FSH alone for prediction of follicular development [83].

In the recently published systematic review of Maheshwari et al., values of likelihood ratios of positive and negative CCCT did not provide sufficient evidence for prediction of nonpregnancy or ART cycle cancellation in 20 eligible studies. Although CCCT was fairly done in a uniform manner, the absence of standardized abnormal test definitions prevented strong conclusions and authors could not support its use as a prognostic tool with the achieved level of evidence [84].

### 4.2. Gonadotropin Analogue Stimulation Test

Identification and measurement of FSH, LH, inhibin-B, and E2 levels flare-up within 24 hours of administration of gonadotropin analogue are the end points of gonadotropin analogue stimulation test (GAST) [85].

Significant positive correlations between ovarian response and the sum of basal and poststimulus inhibin-B levels, or the increase in E2 levels after stimulus, have been demonstrated [86]. Also, McIlven et al. detected a predictive value of elevated E2 levels after GAST for the cancellation of ART cycles among women >39 years of age, with previous poor response or elevated basal FSH [44]. Hendriks et al. also recognized some value of the test for that purpose or as a predictor of pregnancy but reported that it did not show a better clinical performance than the determination of basal inhibin-B or AFC [87].

As a matter of fact, a wide variety of gonadotropin-releasing hormone (GnRH) analogue doses and administration timing, hormones tested in initial and final samples, and their thresholds were not standardized in literature on GAST [84]. Just like the CCCT, GAST should not be an eligible ovarian reserve test in clinical routine, with the present body of evidence.

### 4.3. Exogenous FSH Ovarian Reserve Test

The exogenous FSH ovarian reserve test (EFORT) is based on the increase of E2 and inhibin-B 24 hours after the administration of 300 IU of recombinant FSH (rFSH) on cycle day 3, in order to determine the functional condition of the ovarian apparatus [82].

Increased levels of E2 and inhibin-B after EFORT had been postulated to present the best predictive values for the number of ovarian dominant follicles obtained after stimulus [88], mainly when basal FSH levels were associated with this increase [82]. However, it has been recently demonstrated that, as well as with CCCT and GAST, methodology for EFORT is far from uniform parameters [84] and this lack of pattern maintains the test among those that should be avoided in determining ovarian reserve for infertile patients.

## 5. Ultrasonographic Markers

### 5.1. Antral Follicle Count

Pre-ART ultrasonographic AFC has been shown to be an excellent predictor of ovarian reserve and response, with significant superiority in relation to other markers [89–91]. Studies also demonstrated significant correlations between AFC and commonly performed serum ovarian reserve tests [92], and between AFC and AMH [50, 52]. In a recent systematic review, AFC alone was as accurate as different combinations of clinical, biochemical and other ultrasonographic markers in predicting IVF response [91].

According to the study of Muttukrishna et al., AFC should allow identification of poor response with 89% sensitivity, in spite of low specificity [60], and other authors admit its importance as a screening test for infertile couples [6]. Elgindy et al. assessed AFC with up to 10 mm in mean diameter and found 10.1 ± 3.0 and 5.7 ± 1.0 antral follicles, respectively, for normal and poor responders [64].

According to Maseelall et al., women with AFC ≥ 11 (follicles measuring between 2 and 10 mm present on both ovaries) are more likely to obtain a live birth if compared with those with less antral follicles, who should be advised about the increased risks of miscarriage, cycle cancellation, higher doses of gonadotropins, and fewer oocytes yielded [93]. Despite those results, a recent meta-analysis supported good expectations for women with less antral follicles, since 98.7% of women who got pregnant presented AFC ≥ 4 and cycle cancellation rate among women with at least four antral follicles was 2.5% (95% CI, 1.8–3%) [94].

Special attention has been given to small antral follicles. In the study of Klinkert et al., normal response to stimulus for ART was significantly elevated for women with AFC ≥ 5 units with mean diameter up to 5 mm [29], and Haadsma et al. demonstrated significant correlation between follicles with up to 6 mm and ovarian reserve endocrine tests, in contrast to that observed with larger follicles, which were consistent only with ovarian volume and serum levels of inhibin-B. Also, authors postulated the negative interference of advanced age on small antral follicles count, while the pool of larger units should remain unchanged until the middle of fifth decade of life [92].

Results from literature seem to converge to recognition of the importance of AFC as a predictor of ovarian response. This is why Almog et al. proposed AFC normograms according to age of infertile women [95], which, for instance, still need validation on longitudinal studies, but may offer additional information on protocols individualization strategies.

However, due to 2.6% sensitivity to predict nonpregnancy and 66.7% sensitivity to predict cycle cancellation [94], AFC must not be used as a criterion for ART exclusion, but as a tool for counseling on the low probability of achieving pregnancy and determining individualized treatment protocols in IVF cycles. Since endovaginal ultrasound evaluation is normally performed during the investigation of infertile women, we believe that AFC should be definitely included as a routine test of gonadal reserve in pre-ART evaluation.

### 5.2. Ovarian Volume

Opinions are divided on considering ovarian volume (OV) as an adequate gonadal reserve test. Syrop et al. studied women aged 23–46 years undergoing ART, associating diminished number of oocytes yielded and pregnancy rates with decreased ovarian volumes [96]. Recently, the study of Gibreel et al. observed 92.9% (IC 95%, 86.9–96.7%) and 91.7% (IC 95%, 88.5%–94.2%) specificities for prediction of nonpregnancy and cycle cancellation, respectively, with a 3.0 mL cutoff [94]. Finally, significant correlations had been previously found between reduced ovarian measures, increased age, and elevated serum FSH [97].

However, Elgindy et al. did not observe OV differences between young women with normal and poor response in ART (means of 4.1 ± 0.66 mL and 3.36 ± 0.71 mL, resp.) [64], and McIlven et al. reached similar results when evaluating women at high risk for cycle cancellation [44]. The review of ten well-designed studies on ovarian volume for that purposes concluded that OV presented little applicability in the prediction of poor response or pregnancy [98], as did Broekmans et al. in a previous study [6].

Some studies show concern about the use of OV as a test of ovarian reserve and raised doubts on its reliability being evaluated by two-dimensional ultrasound [99, 100], leading some authors to recommend assessment by three-dimensional ultrasound in order to minimize interobserver variability [101–103]. Thus, OV alone should not be considered as a predictor of ovarian reserve, but we believe that because of its easy performance, it may be included as a routine in diagnostic procedures, adding information to the patient medical records and providing data for further study.

### 5.3. Ovarian Blood Flow

Ovarian blood flow (OBF) has been extensively assessed in natural and stimulated reproductive cycles [104] and negative correlation between age and presovulatory perifollicular blood flow [105]. The study of Shrestha et al. supported such an affirmative by demonstrating high pregnancy rate among women who presented with highly vascularized follicles in early follicular phase [106].

In spite of the foregoing, a recent meta-analysis assessed OBF as a predictor of IVF outcomes, but clinical value was unclear, because of different flow-derived predictors used in literature [94]. This is why ovarian vascular flow may not be used to determine inclusion of infertile couples in ART programs or to infer its results.

## 6. Discussion

The main purpose of ovarian reserve evaluation, especially before ART, is to identify women with poor ovarian reserve for their chronological age. This is why age must always be the first marker to be considered in ovarian reserve assessment. Older women may benefit from ovarian reserve tests since they can help clinicians to find out acceptable chances of pregnancy through IVF. Younger women with altered tests should be first classified as potentially poor responders and must gain benefits from individualized stimulation protocols by quantitative estimative of the pool of FSH-sensitive follicles [6].

The best ovarian reserve test should be able to identify women whose chances of spontaneous pregnancy would be so diminished that it would be appropriate to submit them to therapeutic ovarian stimulation or to identify women whose chances of pregnancy after ART would be so close to zero that it would not be justified to submit them to the potential adverse effects of exogenous gonadotropin stimulus.

Unfortunately, currently available tests do not provide sufficient evidence to be considered ideal and, until new studies provide more consistent results, directly submitting women to ovarian stimulation for an IVF cycle has been shown to be an adequate strategy in order to determine follicular patrimony and female reproductive potential.

Basal ovarian reserve markers (endocrine or ultrasonographic) have definite advantages over dynamic ones, regarding practicability, lower costs, and less side effects. Basal FSH, E2, and inhibin-B have been classically used to infer gonadal function, but AMH and AFC may possibly be the only static markers that can potentially assess the follicular reserve as a whole, in quantity and quality.

Dynamic endocrine tests are, therefore, usually more exceptions than rules in infertile women evaluation, once their routine use involves elevated costs and the potential adverse effects of exogenous stimulation without a direct relation with a real attempt to obtain a pregnancy. Thus, those tests should be reserved for exceptional cases in which the estimated risks of hyperstimulation should carefully be considered.

Since no study succeeded in selecting an ovarian reserve marker with satisfactory sensitivity and specificity alone, multivariate evaluation models have been proposed for a better estimate of functional gonadal capacity. In fact, a recent meta-analysis concluded that several combinations were similar to AFC alone to predict poor response to IVF stimulation [91], but no study considering the combination of AMH and AFC, which are currently being extensively investigated in the evaluation of ovarian reserve, was included. More studies evaluating this combination and others will be necessary to rule out the use of multivariate evaluation models in the assessment of ovarian reserve.

Finally, it is important to point out that ovarian reserve evaluation for specific groups of infertile women, such as those with endometriosis, hyperandrogenic anovulation, autoimmune diseases, or endocrine-metabolic disorders, has been poorly investigated. We have demonstrated a significant reduction in the total number of oocytes and morphologically mature oocytes aspirated after the exogenous stimulus for ART in infertile women with endometriosis compared with controls, in spite of that no significant differences were found in the level of basal AMH, E2, and AFC or in follicular growth after the stimulus [32]. In a recent publication, we have also shown that AMH was the only individual marker with a significant ability for determination of poor response in endometriosis patients with 87.5% sensitivity and 73.7% specificity, for levels ≤1.45 ng/mL [107].

In order to reduce limitations due the scarcity of strong evidence on ovarian reserve evaluation, future research is expected to clarify the predictive value of tests, with special attention to specific diseases related to infertility and multivariate models. For now, ovarian reserve tests shall be used as complementary tools in counseling processes, which must be based on age and individuality. Associated with variables such as the characteristics and conservation of the gametes, the fertilization technique used, seminal quality, embryo conservation and evolution, and the characteristics of the endometrial cavity and of the pelvic-peritoneal microenvironment, ovarian reserve tests may provide the necessary information for the formulation of appropriate stimulation protocols for each couple, reducing both emotional and financial burdens.
